# A transcriptomic and proteomic atlas of expression in the *Nezara viridula* (Heteroptera: Pentatomidae) midgut suggests the compartmentalization of xenobiotic metabolism and nutrient digestion

**DOI:** 10.1186/s12864-020-6459-6

**Published:** 2020-02-06

**Authors:** Shane Denecke, Panagiotis Ioannidis, Benjamin Buer, Aris Ilias, Vassilis Douris, Pantelis Topalis, Ralf Nauen, Sven Geibel, John Vontas

**Affiliations:** 1 Institute of Molecular Biology and Biotechnology, Foundation for Research and Technology – Hellas, N. Plastira 100, GR-70013 Heraklion, Crete Greece; 2 Bayer AG, Crop Science Division, R&D Pest Control, 40789 Monheim, Germany; 3 Department of Crop Science, Agricultural University of Athens, Iera Odos 75, GR-11855 Athens, Greece; 4 Department of Biological Applications and Technology, University of Ioannina, 45110 Ioannina, Greece

**Keywords:** *Nezara viridula*, Southern green stink bug, Transcriptomics, Proteomics, Midgut, P450, Transporter

## Abstract

**Background:**

Stink bugs are an emerging threat to crop security in many parts of the globe, but there are few genetic resources available to study their physiology at a molecular level. This is especially true for tissues such as the midgut, which forms the barrier between ingested material and the inside of the body.

**Results:**

Here, we focus on the midgut of the southern green stink bug *Nezara viridula* and use both transcriptomic and proteomic approaches to create an atlas of expression along the four compartments of the anterior-posterior axis. Estimates of the transcriptome completeness were high, which led us to compare our predicted gene set to other related stink bugs and Hemiptera, finding a high number of species-specific genes in *N. viridula.* To understand midgut function, gene ontology and gene family enrichment analyses were performed for the most highly expressed and specific genes in each midgut compartment. These data suggested a role for the anterior midgut (regions M1-M3) in digestion and xenobiotic metabolism, while the most posterior compartment (M4) was enriched in transmembrane proteins. A more detailed characterization of these findings was undertaken by identifying individual members of the cytochrome P450 superfamily and nutrient transporters thought to absorb amino acids or sugars.

**Conclusions:**

These findings represent an initial step to understand the compartmentalization and physiology of the *N. viridula* midgut at a genetic level. Future studies will be able to build on this work and explore the molecular physiology of the stink bug midgut.

## Background

Insect pests pose a serious threat to food security, which has led to the widespread adoption of transgenic plants expressing insecticidal proteins (e.g. Bt toxins derived from *Bacillus thuringiensis*). These have proved largely effective in controlling chewing insect pests*,* but their success has paved the way for secondary pests, which are not affected by Bt, to become a significant problem [1]⁠. Secondary pests primarily come from the hemipteran order of insects and avoid Bt by feeding on phloem sap or directly on fruit. In particular, stink bug-related crop damage from polyphagous species such as *Halyomorpha halys* (brown marmorated stink bug)*, Acrosternum hilare* (green stink bug), *Euschistus hero* (brown stink bug)*,* and the southern green stink bug *Nezara viridula* have become a major problem [2]⁠. Despite their widespread importance, much still remains unknown about their physiology especially at the genetic level.

**Fig. 1:**
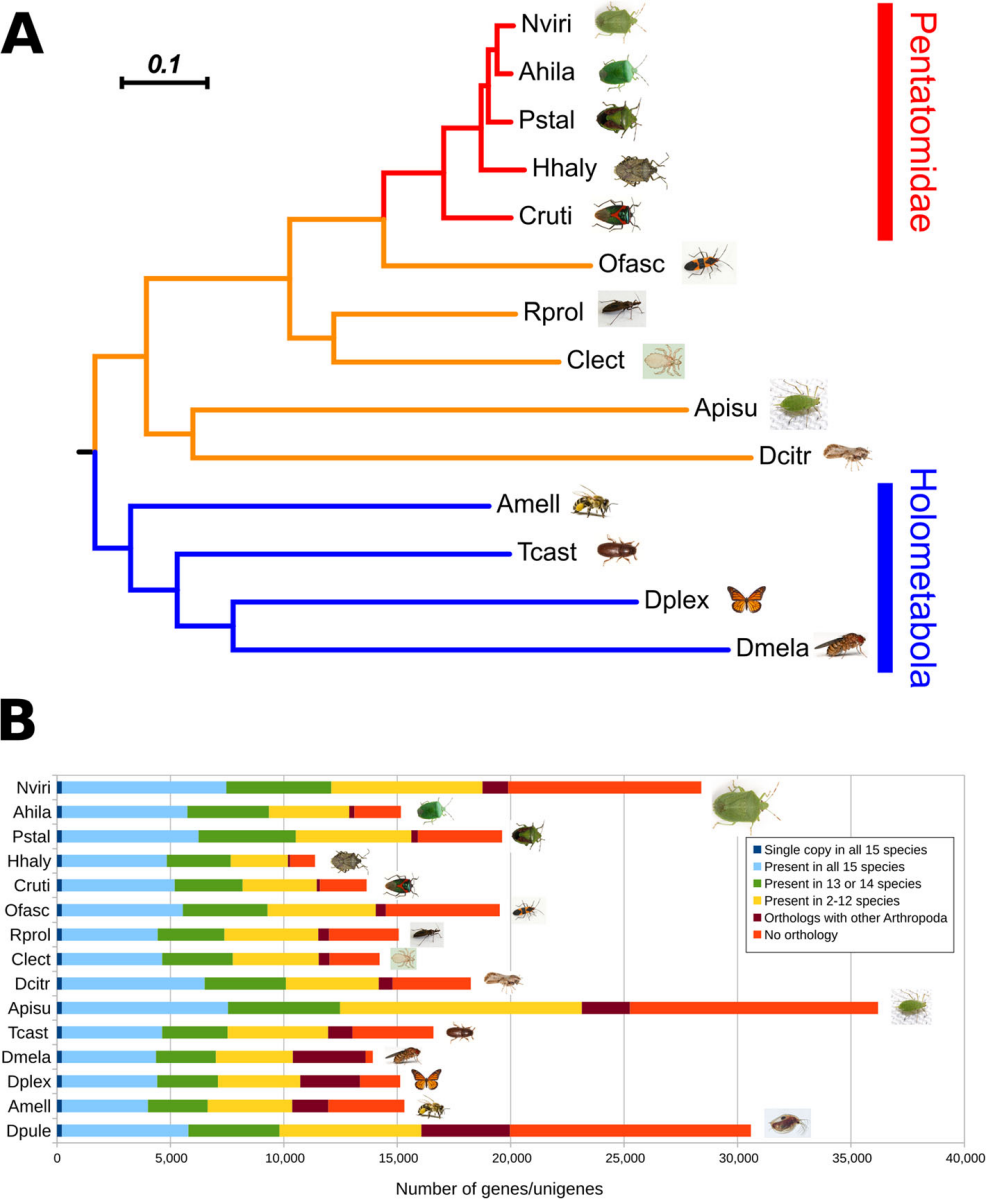
Comparative gene sets among insects. **a** A phylogeny is shown constructed from 221 single-copy genes present in all species included in this analysis. The Pentatomidae (red) form a cluster within the Hemiptera (yellow) order which forms a sister clade to Holometabola (blue). The tree is rooted with the crustacean *Daphnia pulex* (not shown). Black dots indicate nodes with bootstrap support > 75%, whereas gray dots indicate nodes with bootstrap support between 50 and 75%. The scale bar is in substitutions per site. **b** Orthology profile of stinkbugs (names shown in red), compared to other insects. Note the large fraction of species-specific genes in *N. viridula* (Nviri) which is very similar to what has been previously documented for the pea aphid *A. pisum* (Apisu). Species names prefixed with “[T]” indicate that the unigene set was obtained from a transcriptome assembly; for the remaining insect species the data were obtained from a genome assembly. Species names abbreviations: Nviri – *N. viridula*; Ahila – *A. hilare*; Pstal – *P. stali*; Hhaly – *H. halys*; Cruti – *C. rutilans*; Ofasc – *Oncopeltus fasciatus*; Rprol – *Rhodnius prolixus*; Clect – *Cimex lectularius*; Dcitr – *Diaphorina citri*; Apisu – *A. pisum*; Tcast – *Tribolium castaneum*; Dmela – *Drosophila melanogaster*; Dplex – *Danaus plexippus*; Amell – *Apis mellifera*

A tissue of critical importance for insect physiology is the midgut, which interacts directly with the external environment by separating the gut lumen (outside of the body) from the hemolymph (inside the body). Structurally, the insect midgut is composed of a single-cell thick epithelial layer comprised of various cell types including enterocytes involved in absorption/secretion, enteroendocrine cells which produce enteropeptides, and stem cells that can replenish damaged or old cells [3, 4]⁠. Despite these conserved basic features, the insect midgut differs substantially between orders and species ([5]⁠ see Fig. 1 therein). *N. viridula* has a midgut that can be divided into four morphologically distinct sections along its anterior-posterior axis termed M1 (extreme anterior) to the extreme posterior (M4), which is separated from the first three anterior portions by a selective valve [6]⁠. Some physiological roles have been assigned to these compartments; M3 has been implicated in nutrient digestion and the M4 region has long been known to harbor symbiotic bacteria which appear to be essential for growth [7–9]⁠. However, neither the physiological roles or expression profiles of these gut compartments are fully understood.

Principle among the physiological functions of the midgut is the absorption of nutrients. Insects initiate this process through digestive enzymes which breakdown macromolecules, such as proteins and sugars, into oligomers or monomers. Recently, the protease landscape of the *N. viridula* the entire midgut was described by both proteomics and transcriptomics, which revealed an abundance of cysteine proteases in the slightly acidic *N. viridula* midgut [10, 11]⁠. Families of glucosidases thought to metabolize sugar molecules have also been found in the midgut of the pistachio stink bug *Brachynema germari* [12]⁠. The absorption of these smaller molecules has so far not been described in stink bugs, but it can be inferred from other metazoa that they are taken into gut cells via transporter proteins. Several families of transporters have been implicated in amino acid transport, including the Amino Acid and Auxin Permease (AAAP), the Neurotransmitter:Sodium Symporter (NSS), the Amino Acid-Polyamine-Organocation (APC), and the Proton-dependent Oligopeptide Transporter (POT/PTR). Other groups, such as the Sugar Porter (SP), the Solute:Sodium Symporter (SSS), and the SWEET families have been implicated in sugar transport [13]⁠. Different labs have adopted different nomenclatures for these transporters (see Additional file 5: Table S1), but this has not prevented several groups from identifying members of these transporter families in insects [14–16]⁠, although this has not been performed in any stink bug species.

While the midgut must actively absorb nutrients from the diet, it must simultaneously form a barrier that selectively excludes toxic xenobiotics such as plant secondary metabolites or insecticides [17]⁠. One of the key mechanisms of regulating the penetration and toxicity of such molecules is through metabolism by xenobiotic-metabolizing enzymes such as cytochrome P450s (P450s), carboxylesterases, and glutathione-S transferases. Members of these families are present in the gut and chemically modify xenobiotics, which limits their uptake and often results in their detoxification. P450s are particularly well studied; upregulation of P450s in the midgut has often been found to underpin insecticide resistance [18–20]⁠. Information on P450s in stink bugs is currently limited to one species *Halyomorpha halys,* where a preliminary identification and analysis has been performed [21, 22]⁠.

In order to better understand the genetics and physiology of the midgut of the southern green stink bug *N. viridula,* we performed a detailed characterization of transcript and protein expression along the anterior-posterior axis. The unigene set obtained from the transcriptome assembly included the vast majority of conserved insect genes, allowing for a large scale phylogenomic analysis that placed *N. viridula* as a sister species to the green stink bug *A. hilare*, with high confidence. Moreover, the filtered unigene set was used for an orthology analysis, by comparing *N. viridula* to other stink bugs as well as other hemipteran and holometabolan insects suggesting an increased fraction of species-specific genes. We further examined *N. viridula* gut physiology by identifying gene families and GO terms enriched in specific midgut compartments, concluding partially overlapping roles for different sections of the midgut. This detailed profiling of stink bug midgut expression should serve as a basis for more detailed molecular characterization of stink bug midgut physiology in future studies.

## Results

### Overview of Transcriptome and proteome

The four midgut sections of adult *N. viridula* individuals were dissected and each of these tissues were sequenced together with the corresponding carcass in four biological replicates yielding a total of 1,426,685,586 reads. These were assembled de novo into 314,260 transcripts (Table 1), and running TransDecoder on this transcript set predicted a total of 73,752 peptides. This peptide set was used as the theoretical database to identify proteins from gel-free proteomics in each of the four midgut compartments, and resulted in a total of 3472 unique proteins in our samples (Table 1). No differences in terms of the enrichment of membrane proteins were observed between the supernatant and pellet fractions of the proteomic analysis (Additional file 6: Table S2). Lastly, we tested whether the presence/absence of a protein in the proteomics set was associated with its expression in the transcriptome and found that proteins identified in the proteome showed on average far higher expression values, compared to the non-detected proteins (Additional file 1: Figure S1). Full tables showing the expression levels reported in transcripts per kilobase million (TPM) and presence or absence in proteomics are reported in Additional file 7: Table S3 and Additional file 8: Table S4, respectively.

**Table 1:** Statistics of transcriptome and proteome: An overview of the transcriptome and proteome is given in terms of total reads, contigs, unigenes, and detected proteins

Transcriptome				
	Total Reads	Total Contigs	Total unigenes	Total non-bacterial unigenes
	1,426,685,586	314,260	28,402	25,890
Proteome with bacterial-like transcripts				
Total M1	Total M2	Total M3	Total M4	Total proteins
2401	1992	2472	2370	3472
Proteome without bacterial-like transcripts				
Total M1	Total M2	Total M3	Total M4	Total proteins
2377	1968	2102	1945	3027

In order to perform a phylogenomic analysis, the *N. viridula* protein set was filtered to 28,402 unigenes by grouping transcripts at the gene level using the Trinity accession numbers, which yielded superior BUSCO scores (Additional file 2: Figure S2). This gene set was compared to publicly available genomes and transcriptomes from stink bugs and other insects (Additional file 9: Table S5). More specifically, we used the standalone version of the orthology database OrthoDB v9 [23]⁠ to obtain a list of 221 single-copy genes present in all species, which we subsequently used for a phylogenomic analysis. This analysis showed that all stink bugs clustered together and formed a monophyletic clade, as they all belong to the Pentatomidae family of Hemiptera (Fig. 1a). The phylogeny was complemented by an orthology analysis, in order to compare gene copy number across various insect lineages (Fig. 1b). Interestingly, the unigene set for *N. viridula* contained a large number (*n* = 8510) of unigenes that have no ortholog with other arthropod species. This number is elevated in *N. viridula* even when compared to the pentatomid stink bug *P. stali* that was analyzed using the same Trinity-based pipeline. The majority of these genes (*n* = 5927) has a BLAST match (e-value < 1e-05) in the Uniref50 database, with almost half of them (*n* = 2378) being similar to an arthropod protein (Additional file 3: Figure S3). Of the 2583 genes that do not have a BLAST match in Uniref50, 1757 are transcribed with a TPM value > 1, in at least one of the four midgut compartments, indicating that the corresponding genes should be further studied to determine whether they are functional.

It should be noted that a considerable fraction of the *N. viridula* unigene set are similar to bacterial proteins (*n* = 2512). These genes most probably originate from the bacterial symbionts associated with *N. viridula*. There was a significant difference in the mean transcriptional level of the gut regions, for 871 of them (one-way ANOVA tests using the log-transformed TPMs) with the vast majority being up-regulated in the M4 gut region, which harbors the bacterial symbionts in pentatomid stink bugs [9, 24]⁠. Most of these M4-specific genes originate from γ-proteobacteria, which is in agreement with previous studies [9, 25]⁠. Another set of genes appears as being expressed in the M1 and M2 regions only. Interestingly, their taxonomic profile differs from the previous ones, because their majority originates in the Bacteroidetes/Chlorobi clade. As this study was aimed at analyzing the midgut of *N. viridula* these 2512 bacterial-like genes were filtered out of the unigene set and all subsequent analysis was done on the set of 25,890 remaining eukaryote-like unigenes.

### Analysis of functions in each gut compartment

**Fig. 2:**
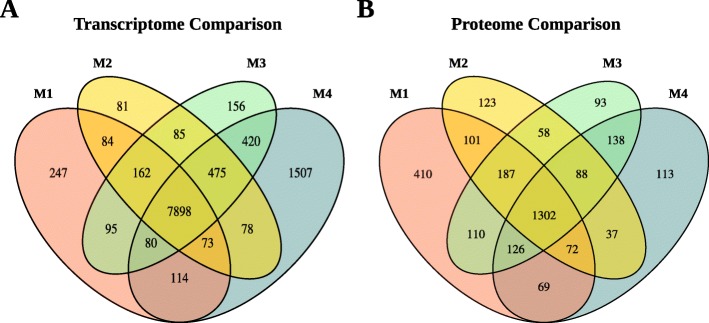
Shared transcript and protein expression. Venn diagrams are shown for both detected transcripts (**a**) and proteins (**b**), showing expression > 1 transcript per million (TPM) in each tissue. In each case, a sizable portion of the detected features are found across all midgut compartments, indicating a that many genes are expressed across the anterior-posterior axis. In both cases the M1 and M4 region display the most distinctiveness. The relatively lower number of proteins detected in the proteome compared to transcripts in the transcriptome is reflective of the sensitivities of these two technologies

**Fig. 3:**
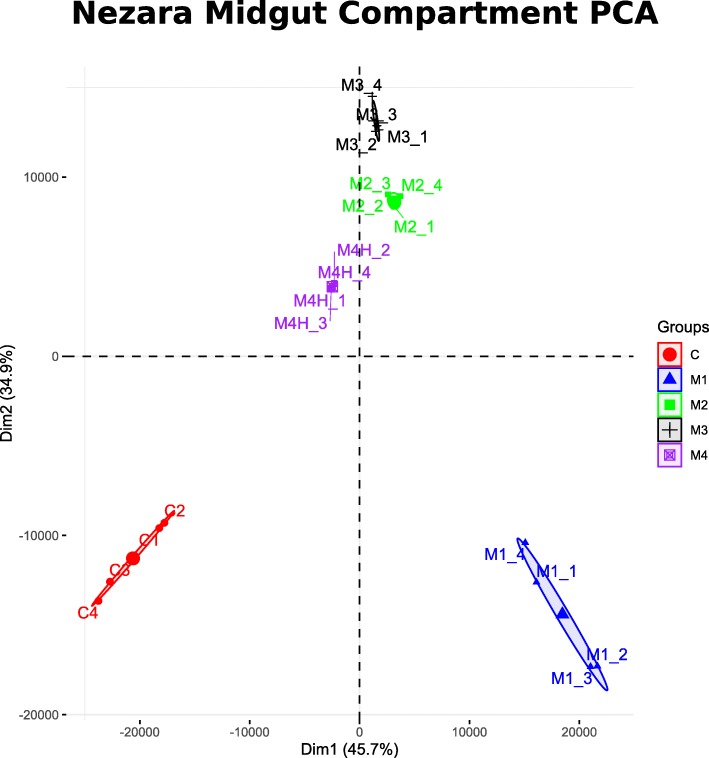
Principle Component Analysis. The results of a principle component analysis of the expression of all unigenes is shown. The first two principle components explain a total of 78% of the total variation detected within the RNA-seq data. Each shape and color represent a distinct sample (M1: Blue triangle, M2: Green square, M3: Black cross, M4: purple crossed square, carcass: red circle). The variation in each sample is shown with an ellipse which encompass all replicates in that sample

In order to obtain an overview of the expression profile along the midgut, transcripts expressed > 1 TPM and proteins detected with gel-free proteomics along the *N. viridula* midgut were compared visually with Venn diagrams (Fig. 2). Despite the obvious morphological differences of these segments, the majority of transcripts (68%; *n* = 7898) and a significant amount of proteins (43%, *n* = 1302) were present in all compartments. In both analyses the M1 and M4 regions had the highest number of genes or proteins detected in only one compartment. To further explore the broad differences between midgut compartments we also performed a principle component analysis (PCA). The first two dimensions of the PCA explained 45.7 and 34.9% of the variation respectively (Fig. 3). All biological replicates in a sample clustered together, which is indicative of relatively high reliability of the tissue sampling. Also of note is the relative clustering of the M2, M3, and M4 sections especially along the first principle component, suggesting that these samples show similar transcriptome profiles. Unsurprisingly, the carcass sample clustered independently, but so also did the M1 section of the midgut suggesting that it has a distinct transcriptional profile to the other midgut sections. Collectively, these data suggest that while most genes detected in the analysis were commonly shared among all compartments, M4 and especially the M1 appear the most distinct.

**Fig. 4:**
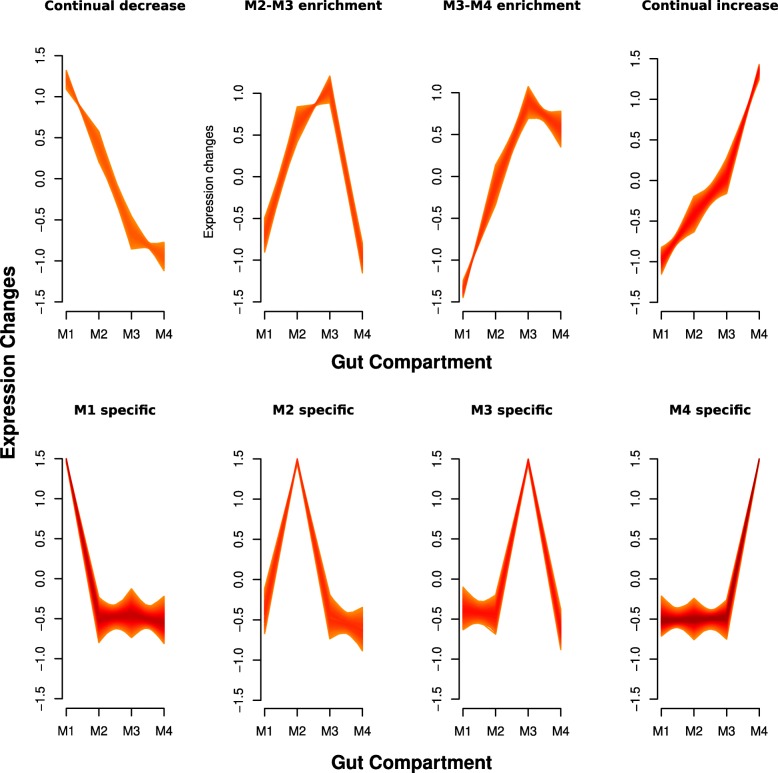
Gene expression patterns along the midgut. The results of the fuzzy-C means clustering is shown. Transcripts were grouped into eight categories based on their relative expression pattern, and all members with membership values > 0.6 were plotted. The darker shading on the plot indicates a larger number of individual transcripts which show that expression pattern. The top four clusters are composed of more complicated patterns, whereas the bottom four clusters display transcripts enriched specifically in one compartment

A more detailed understanding of each midgut compartment was obtained by identifying groups of transcripts and analyzing them for enrichment in family membership (Pfam) or gene ontology (GO) terms. Fuzzy C-means clustering yielded eight groups of genes which displayed differing expression patterns along the midgut (Fig. 4). Four out of the eight clusters reflected transcripts specific to a single compartment. The remaining four clusters showed more complex patterns of expression along the gut. For example, one cluster showed transcripts which gradually increased in expression level from anterior to posterior (M1 < M2 < M3 < M4). The 500 most highly expressed genes were also grouped from each compartment in order to estimate the predominant function of each section. These analysis yielded 12 groups of genes (8 clusters and 4 Top500 groups) which were analyzed in bulk by looking for enriched gene families and GO terms.

The M1-M3 region tended to display similar arrays of enriched protein families and GO terms with regards to both specificity and overall expression level. In the M1-M3 compartments families like cysteine proteases or GO terms related to proteolysis were found significantly enriched in both the top 500 most highly expressed genes and in the compartment specific cluster (Table 2; Additional file 10: Table S6). Likewise, families associated with xenobiotic metabolism (P450s, carboxylesterases) or GO terms associated with these reactions (oxidation-reduction process) were frequently found in the anterior sections. In contrast, the M4 displayed GO terms relating to transmembrane transporter proteins and an enrichment in proteins from the sugar porter family (PF00083; Table 2; Additional file 10: Table S6). Of all of the other clusters containing genes with more complex expression patterns, only one (M1 < M2 < M3 < M4) showed a significant enrichment in any GO term or family; the zinc finger C2H2 family were overrepresented in this fuzzycluster. From the GO term and Pfam enrichment analysis it can be inferred that the anterior portion of the midgut (M1-M3) has a predominant role in metabolism of xenobiotics and nutrients, while the posterior has a role in the transport of nutrients.

**Table:** **Table 2** A table is shown reflecting all of the significantly enriched GO terms from the gene groups produced from either the Fuzzy C-means clustering or the Top 500 most highly expressed genes in each sample. For each GO term, supporting information is provided such as the Gene Group from which it was identified the Code name, False discovery rate and information about how many genes with this term were identified compared to the total number in the transcriptome

Gene Group	Code Name	False Discovery Rate	# of Genes in Group with Term	# of Genes in Transcriptome with Term	GO Term Annotation
Continual_increase_fuzzycluster	PF13909	0.000130095	23	189	C2H2-type zinc-finger domain
Continual_increase_fuzzycluster	GO:0003676	0.000187859	69	1115	nucleic acid binding, molecular_function
M1_fuzzycluster	GO:0006412	1.39345E-06	52	548	translation, biological_process
M1_fuzzycluster	PF02958	1.62758E-06	19	85	Ecdysteroid kinase
M1_fuzzycluster	PF00112	0.000255183	17	110	Papain family cysteine protease
M1_fuzzycluster	PF00504	0.000255183	11	43	Chlorophyll A-B binding protein
M1_fuzzycluster	GO:0008234	0.000504127	17	121	cysteine-type peptidase activity, molecular_function
M1_fuzzycluster	GO:0006414	0.000780785	11	56	translational elongation, biological_process
M1_Top 500	PF00112	1.58324E-11	19	110	Papain family cysteine protease
M1_Top 500	GO:0008234	2.43945E-11	19	121	cysteine-type peptidase activity, molecular_function
M1_Top 500	PF00135	1.63518E-08	15	100	Carboxylesterase family
M1_Top 500	PF02958	1.13244E-06	12	85	Ecdysteroid kinase
M1_Top 500	PF00067	4.40651E-06	14	146	Cytochrome P450
M1_Top 500	GO:0055114	0.000163275	37	1078	oxidation-reduction process, biological_process
M1_Top 500	GO:0006508	0.000818748	18	394	proteolysis, biological_process
M2_fuzzycluster	PF00112	5.48524E-05	11	110	Papain family cysteine protease
M2_fuzzycluster	GO:0008234	0.000114662	11	121	cysteine-type peptidase activity, molecular_function
M2_Top 500	GO:0055114	3.88423E-08	53	1078	oxidation-reduction process, biological_process
M2_Top 500	PF00112	2.93311E-07	15	110	Papain family cysteine protease
M2_Top 500	GO:0008234	7.96227E-07	15	121	cysteine-type peptidase activity, molecular_function
M2_Top 500	PF00067	9.31946E-07	16	146	Cytochrome P450
M3_Top500	GO:0055114	1.54695E-07	51	1078	oxidation-reduction process, biological_process
M3_Top500	PF00067	4.50598E-05	14	146	Cytochrome P450
M3_Top500	PF00135	4.50598E-05	12	100	Carboxylesterase family
M4_fuzzycluster	GO:0055085	1.64321E-13	137	572	transmembrane transport, biological_process
M4_fuzzycluster	GO:0016021	1.03943E-10	122	543	integral component of membrane, cellular_component
M4_fuzzycluster	PF12796	2.91941E-05	83	387	Ankyrin repeats (3 copies)
M4_fuzzycluster	GO:0008061	4.19023E-05	29	85	chitin binding, molecular_function
M4_fuzzycluster	GO:0016020	5.10354E-05	95	517	membrane, cellular_component
M4_fuzzycluster	PF00083	0.000120073	36	117	Sugar (and other) transporter
M4_fuzzycluster	GO:0005515	0.000164315	307	2221	protein binding, molecular_function
M4_fuzzycluster	GO:0006468	0.000464805	57	282	protein phosphorylation, biological_process

### Identification and analysis of detoxification enzymes and nutrient transporters

**Fig. 5:**
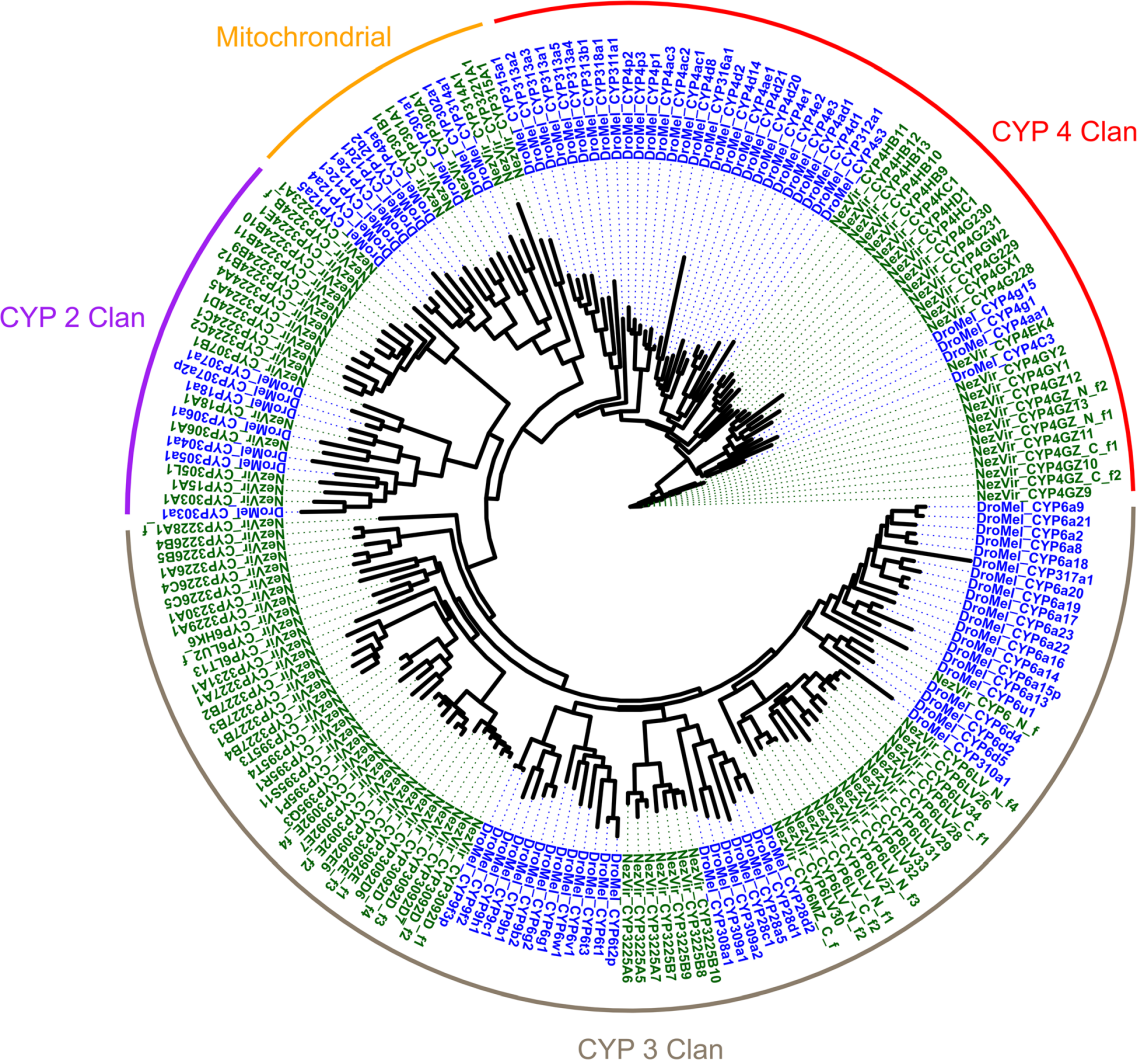
Phylogeny of identified P450s in *N. viridula.* A phylogenetic tree constructed of *N. viridula* P450s is shown along with other reference species. The names of each of the four CYP clans is shown at the outer rim. Individual P450s used in the tree were those *N. viridula* P450s identified in the current study (black), or P450s from *D. melanogaster* (dark red)

The enrichment of P450s in the anterior region of the midgut led us to annotate individual members of this gene family using a pipeline centered around homology searches. Testing our pipeline on several well-annotated proteomes, suggested that our method predicted a number of P450 genes that was close to those previously reported in the literature for other insects (Additional file 11: Table S7). After manually combining P450 fragments which displayed overlaps and removing contaminants, a total of 109 P450s were identified in our *N. viridula* unigene protein set (Fig. 5; Additional file 15; Additional file 12: Table S8). The expression profile of these P450s was then analyzed by family to observe any compartmentalization of functions. Of particular interest was the CYP6 family, which has a known role in insecticide metabolism [18]⁠ and showed high expression across all midgut compartments in our dataset with a clear enrichment in the anterior portion of the midgut (M1-M3) compared to both the M4 region and the carcass. Also of note were five CYP4G genes that are commonly implicated in cuticular hydrocarbon biosynthesis [26]⁠. All four of these genes in *N. viridula* showed high levels of expression only in the carcass sample (Additional file 12: Table S8). Averaging the expression of all P450s, there was roughly twice the expression in the anterior portions of the midgut compared to the posterior section.

The enrichment of transporter proteins in the M4 region of the midgut was expanded further by identifying individual members of several families of sugar and amino acid transporters using an in house pipeline (see Materials and Methods). Sugar transporters belonging to the SP, SSS, and SWEET families were identified and analyzed for their expression pattern along the midgut (Additional file 16; Additional file 13: Table S9). The 11 SSS transporters that were identified, were expressed at very low levels in all midgut compartments. Only two SWEET transporters were detected, one of which showed high expression and 2–4 fold enrichment in all midgut compartments compared to the carcass. However, by far the largest group of sugar transporters was the SP family with 84 detected transporters. This group was incredibly diverse in its expression pattern; different SPs showed specificity or enrichment in different midgut compartments. However, in accordance with the Pfam enrichment of sugar transporters in the M4 region (Table 3), the highest total expression and the largest number of highly expressed genes (> 50 TPM) were found in the M4 region of the midgut (Table 3).

**Table 3:** Nutrient transporter expression in the midgut

Measuremnet	POT	APC	AAAP	NSS	SWEET	SSS	SP
Average M1 Expression (TPM)	32.73	12.60	22.67	2.60	56.08	0.84	36.89
Average M2 Expession (TPM)	103.55	18.06	49.53	2.08	46.17	0.26	36.73
Average M3 Expression (TPM)	152.57	17.88	30.03	2.01	40.04	0.70	26.94
Average M4H Expression (TPM)	33.04	48.00	85.90	9.07	34.44	5.17	54.84
Average Carcass Expression (TPM)	2.98	15.86	30.02	3.90	15.23	19.24	16.54
# of M1 Transcripts over 50 TPM	1	1	1	0	1	0	9
# of M2 Transcripts over 50 TPM	1	1	2	0	1	0	7
# of M3 Transcripts over 50 TPM	1	1	1	0	1	0	4
# of M4H Transcripts over 50 TPM	0	3	6	0	1	0	16
# of Carcass Transcripts over 50 TPM	0	0	1	0	0	1	4

After filtering for transcripts with <1 TPM in all compartments, average expression values were displayed along with the total number of highly expressed > 5 and > 50 > 50TPM genes for each transporter family in each compartment

**Fig. 6:**
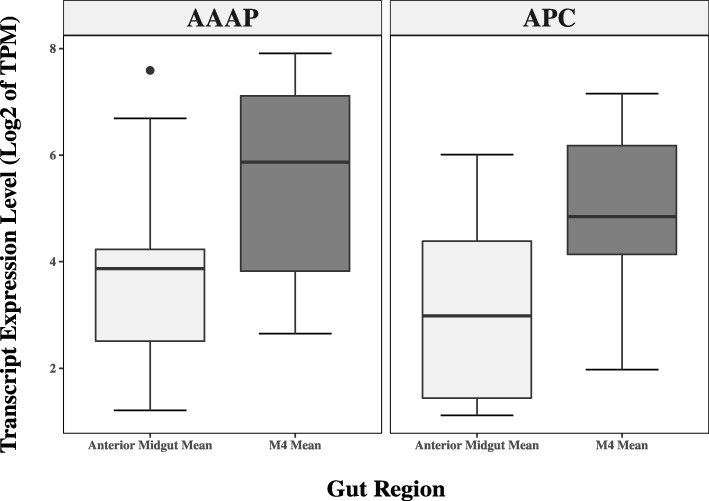
Enrichment of AAAP and APC transporters in the M4 midgut region. A boxplot is shown comparing the expression of all AAAP and APC transporters showing along the midgut. Only genes showing expression above >1TPM in either the M4 region or the anterior region (average of the M1-M3 region) were plotted. The M4 region of the gut displays an enrichment in both APC and AAAP expression compared to the anterior section of the midgut

Amino acid transporters belonging to the families NSS, APC, POT, and AAAP families were all represented by at least four members in *N. viridula* (Additional file 16; Additional file 13: Table S9). The ten NSS family members generally showed low expression, and only one NSS showed expression values of > 10 TPM. The five POT family members showed a similar low expression apart from DN111091_c2_g2, which showed very high (> 200 TPM) expression in the M2 and M3 regions of the midgut. The APC and AAAP families were larger, with 18 and 15 members respectively. Furthermore, the number of transcripts from both APC and AAAP showing very high (> 50 TPM) expression was elevated in the M4 tissue (Table 3); 3/15 AAAPs and 6/18 APCs were highly expressed in the M4 region. Lastly, the expression of these families in the M4 (APC: 48.00 ± 15.4, AAAP: 85.9 ± 26.8) and was higher than the average anterior midgut expression (APC: 16.2 ± 7.0: AAAP: 34.1 ± 17.8; Fig. 6).

## Discussion

Stink bugs are an emerging threat to food security but are still poorly understood at the genetic level. Here, we aimed to provide basic genetic and physiological knowledge about the southern green stink bug *N. viridula* through RNA-seq and proteome sequencing with a strong focus on the midgut.

### Orthology and phylogeny

Apart from specific information regarding midgut physiology, the completeness of our transcriptome (Additional file 2: Figure S2) allowed for an orthology analysis that included another three stink bug species with a publicly available genome or transcriptome (Fig. 1a, b). First, based on single-copy genes, this analysis showed that *N. viridula* is a sister species to *A. hilare*. Additionally, a high number of unigenes unique to *N. viridula* was identified compared to the other insects included in the comparison (Fig. 1b). Of course, this finding could be an artifact from the inherent limitations of de novo assembly of RNA-seq data. However, it should be noted that for three stinkbug species in this study (*A. hilare*, *C. rutilans*, and *P. stali*) de novo transcriptome assemblies were also used, but did not yield such high numbers of unique genes. While many of these genes are bacterial-like or are similar to transposable elements, excluding them still yields a large number of genes unique to *N. viridula.* Such lineage-specific genes are worth of further investigation because they might be associated with the particular ecological characteristics of the species.

A more detailed analysis of the bacterial symbionts is definitely needed, especially because they are essential for the survival of their host [9]⁠. However, since our RNA extraction protocol was based on polyA selection, it specifically discarded the vast majority of bacterial transcripts. As a result, we hesitate analyzing further these bacterial genes because such an analysis will have a reduced value, being based on an incomplete bacterial set of genes.

### The anterior midgut: M1-M3

The anterior portions of the midgut (M1-M3) showed similar functional profiles according to the analysis performed in the current study. When considering groups of genes specific to a certain compartment or when selecting the most highly expressed genes, varying combinations of xenobiotic metabolizing enzymes (P450s and carboxylesterases), and cysteine proteases were found. This largely agrees with what has previously been shown for stink bug midguts in non-genetic studies; multiple groups have found that cysteine proteases predominate in the stink bug midgut and that their activity is enriched in the M1 and M3 regions [7, 10, 21, 27, 28]⁠. Interestingly, the fact that enrichment in these proteases was found in multiple compartment-specific clusters of genes (Table 2) suggests that different individual members of these groups may be upregulated in different compartments. A further functional investigation of these sections of the midgut is needed, especially the M1 section. While possessing many of the same GO terms as the M2 and M3 section, this compartment had the most number of unique proteins detected and clustered apart from the M2 and M3 regions in the PCA presented here and in a parallel study considering the midgut transcriptomes of *N. viridula* midgut sections [29]⁠.

In order to gain a more detailed understanding of this compartmentalization of the M1-M3 region, we identified individual members of the cytochrome P450 family. Our analysis found a total of 109 P450s, significantly less than the > 160 found in a closely related stink bug *Halyomorpha halys* [21]⁠. However, this may be an artifact of the identification pipeline used in this study which predicted only 123 P450s in *H. halys* (Additional file 11: Table S7). Of particular interest are P450s that could cause insecticide resistance. More than 18% (20 / 111) of P450s detected were in the CYP6 family known to have roles in insecticide metabolism in the midgut of other species [18, 20]⁠. Additionally, these genes tended to have higher absolute expression in the midgut and were enriched in the M1-M3 regions compared to the M4 region or the carcass (Additional file 12: Table S8). The homology of these to known drug metabolizing enzymes and expression in a known metabolic tissue would place them on a shortlist of candidates for functional characterization of xenobiotic metabolism in vivo or in vitro.

### The posterior midgut: M4

Previous physiological studies on the stink bug midgut have dealt primarily with the M4 region of the midgut, focusing in particular on symbiont-host interactions. The difficulty of gene manipulation in stink bugs has led to a focus on identifying bacterial factors that mediate symbiosis [6]⁠. Some recent efforts have been made to understand host factors in the bean bug *Riptortus pedestris*, but differences in morphology between this insect and *N. viridula* (a bulb in the M4 region which appears to have specific properties), prevents a direct comparison with *N. viridula* [30, 31]⁠.

The identification of nutrient transporters in the current study showed an enrichment of certain amino acid transporter families (APC and AAAP) in the symbiont-containing M4 region (Fig. 6). While the exact role of the *N. viridula* symbionts has not been established, at least some of them appear to be essential for host survival [9]⁠, and the basis of this survival is thought to be in part due to the synthesis of essential amino acids [32]⁠. Furthermore, the presence of symbionts in stink bugs is highly correlated with herbivory [8]⁠, suggesting that provision of essential amino acids underpins the fitness advantage conferred by these bacteria. It is thus tempting to speculate that increased expression of such transporters helps mediate the symbiotic interaction between bacteria and *N. viridula* as has been shown in the aphid-*Buchnera* symbiosis [32]⁠. However, stink bugs lack aphid bacteriocytes (where these transporters have been localized to) and have a more promiscuous relationship with bacteria, so this comparison is not perfect. The role (or lack thereof) of these proteins in interacting with symbiotic bacteria awaits functional characterization.

## Conclusions

In order to understand the function of an organism, tissue, or cell, one must first know the landscape of genes and proteins that are present there. While this study is purely descriptive in its nature, knowledge of the protein landscape of a tissue can provide a useful resource on which functional studies can build. This has been true in *D. melanogaster* where tissue-specific (FlyAtlas [33];⁠ or intra-gut [19, 34, 35]⁠ expression atlases have been used to address research questions beyond the scope of their original purposes. In addition to the atlas provided here, a parallel study (published while this manuscript was under review) has also sequenced several portions of the *N. viridula* midgut, which provides opportunities for comparisons and cross-validation [29]⁠. Lastly, the recent validation of efficient RNAi in both *N. viridula* nymphs and adults, means that these resources can begin to be used to probe the molecular physiology of the stinkbug midgut.

## Methods

### Insects and dissections

A population of *N. viridula* was obtained from Bayer and bred for several generations at the Institute of Molecular Biology and Biotechnology (Heraklion, Greece). Adults were maintained on 12 h light:dark cycles at a temperature of approximately 23 °C in mesh cages (Bugdorm, Taiwan). All individuals were raised in these cages with a mixture of organic sunflower seeds, carrots, and broad beans for food.

For the transcriptomic analysis, the four sections of the midgut (M1-M4; Additional file 4: Figure S4) were dissected from *N. viridula* adults in four biological replicates. Each biological replicate consisted of a single section of the gut take from at least 10 individuals, so RNA from 40 total midguts was sent for sequencing. The microscopic hindgut was included with the M4 region due to the inability to effectively separate these two tissues. The remaining non-midgut carcass was also collected and sequenced for comparative purposes. All tissues were dissected in RNAse-free phosphate-buffered saline (PBS) on ice, and RNA was extracted with the GeneJet RNA purification kit (Thermo Scientific) according to the manufacturer’s instructions. Subsequently, all midgut samples were sent for strand-specific, paired-end sequencing using the Illumina HiSeq 2500 platform with 100 bp per read (Macrogen Sequencing facility, Seoul).

For the proteomic analysis, the same four midgut parts were dissected and processed in three biological replicates by gel-free proteomic analysis at the Centre for Proteomics (Antwerp, Belgium) as was described elsewhere [36]⁠. Each sample was split into membrane and supernatant protocols to in order to enrich for membrane bound vs soluble proteins respectively, and detected proteins from from both membrane and soluble fractions were pooled in order to generate a more complete protein set for each tissue. Finally, proteins were identified by the proteomics facility using a theoretical database which included the predicted peptides from the Trinity-based transcriptome assembly (see next section in Methods) and were further filtered so that we only obtain high-quality predictions by setting the threshold for the protein identification probability at > 95%.

### Transcriptome processing and annotation

Raw RNA-seq data were obtained in FastQ format and checked for quality with FastQC [37]⁠. Assembly of reads was accomplished with Trinity v2.5.1 [38]⁠ using parameters for strand specific orientation (−seqType fq --SS_lib_type RF). Both the nucleotide assembly and raw reads were deposited under the bioproject number PRJNA557118A corresponding protein set was generated using TransDecoder [39]⁠ with the default parameters. The resulting set of proteins was filtered in order to obtain a unigene set (one protein from each gene). To achieve this we made use of the Trinity naming scheme as it is encoded in the fasta accessions (e.g. TRINITY_DN1000_c115_g1_i1) and only kept the longest protein of each gene (“g”) level as recommended by the Trinity manual. Finally, CD-HIT [40]⁠ was used to further filter this reduced protein set, by removing proteins that are entirely contained within larger proteins with the default word size and a sequence identity threshold of 1.0. Annotation of proteins and transcripts was accomplished by a combination of BLAST searches against the Uniref50 database [41]⁠ and protein domain identification with InterProScan [42]⁠. Proteins having a significant similarity (e-value < 1e-05) to plant or bacterial sequences were discarded and not taken into account in downstream analyses.

Expression values for each unigene were obtained with Kallisto [43]⁠, which yielded normalized read counts in TPM. The mean TPM value for each transcript in each compartment was calculated by averaging the expression across the 4 biological replicates and was then used to perform comparisons of midgut function. Fuzzy C-means clustering was accomplished using the Mfuzz R package [44]⁠ which grouped all transcripts into eight clusters based on their expression pattern along the gut. All transcripts showing strong membership with a particular cluster (α values > 0.6) were kept for further analysis. In parallel, the predominant function of each gut compartment was estimated by selecting the 500 most highly expressed genes (*Top500*) from each midgut compartment. All groups of genes from the Fuzzy C-means clustering and Top500 analysis were searched for their enrichment in gene families (Pfam) or GO terms using a custom R script implementing Fisher’s exact test with a minimum number of genes, set at 10 and a false discovery rate of < 0.001. The principle component analysis was accomplished by using the *fviz_pca* function in the *factoextra* R package.

### Phylogenomic analysis

For the phylogenomic analysis we first mapped the above-mentioned *N. viridula* unigenes onto the ortholog groups of OrthoDB v9 [23]⁠, at the Arthropoda node. Additionally, we downloaded and processed the transcriptomes of another three stink bugs so that we only keep one isoform per gene; the brown-winged green stink bug *Plautia stali* [45]*⁠, Chalcocoris rutilans* [46]⁠, and the green stink bug *A. hilare* [46]⁠. These processed transcriptomes were then mapped on OrthoDB. Subsequently, we used custom Perl scripts to find single-copy genes that were present in all stink bugs and also other insects covering some of the main insect orders. A custom RAxML-based pipeline was used for reconstructing the phylogeny, with the crustacean *Daphnia pulex* as the outgroup. In order to compare unigene sets between species, custom Perl scripts were used to identify different orthology profiles (single-copy in all species, present in all species, etc) in the selected insect species.

### Identification of P450s and nutrient transporters

All P450s found in our dataset, were identified based on several criteria common in the literature. Briefly, a curated P450 fasta file containing sequences from *Drosophila melanogaster, Apis mellifera,* and *Bombyx mori* was downloaded and cleaned from the Cytochrome P450 homepage ([47]⁠; www.p450-homepage.com). These genes were used as a query to search the *N. viridula* unigenes reported here using BLAST with an e-value cutoff of 1e-3 and a query coverage of > 40%. Unique hits from this list were then reciprocally searched with BLAST against the curated P450 fasta file, using the same parameters and were filtered for amino acid lengths of between 150 and 650 amino acids. These candidate proteins were further, further selected by presence of a PFAM P450 domain (PF000067). All P450s with overlapping sequence fragments were joined, and all P450s named in accordance with the established P450 nomenclature by Dr. David Nelson. A custom RAxML based pipeline was used to generate the phylogenetic tree using a maximum likelihood approach and 500 bootstraps. All *N. viridula* and *D. melanogaster* P450s were used in the tree, and visualization was accomplished using ggtree [48].

The identification of nutrient transporters is not fully standardized in insects. As all of these transporters are members of the SLC superfamily, a strategy was adapted from a previous publication [49]⁠ which identified novel SLC members in distantly related species. Human sequences from each transporter family were compiled from Bioparadigms (http://slc.bioparadigms.org/) and *D. melanogaster* family sequences were taken from the Flybase solute carrier gene groups [50]⁠ supplemented with manually curated additions. In each species, a HMM database was constructed for several sugar and amino acid transporter families (Additional file 5: Table S1) which were then used to search our *Nezara* protein sequences using the HMMer package [51]⁠. Candidate transporters were further filtered by reciprocal blast and were considered to belong to a family as long as i) The top hit was in a particular family ii) 4 out of 5 of the top hits were in in that same family and iii) the query showed at least 20% similarity to one other member of that family. In cases where there were less than five predicted family members, it was sufficient that all members were in the top 5. Further filtering was done based on the presence of at least three transmembrane domains predicted by TMHMM [52]⁠. In order to remove any abnormally short or long members of gene families, sequences were removed which had protein lengths more than one standard deviation from the minimum or maximum length of the corresponding protein family in humans and *Drosophila* which was modified from the filtering methodology of OrthoDB [23]⁠. Candidates discovered using either human or *D. melanogaster* databases were then used to search the *N. viridula* unigene protein set iteratively.

## Supplementary information

**Additional file 1: Figure S1.** Comparison of transcript expression with protein detection. A boxplot comparing the output of the RNA-seq analysis with the proteomic analysis is shown. All unigenes were categorized as either “Present” or “Absent” in the proteome (x axis) and plotted according to their transcription level (y axis) in terms of log (base 2) transformed TPM value. These data suggest that genes detected in the proteome had generally higher transcript expression compared to those proteins that were not detected.

**Additional file 2: Figure S2.** Quality of the *N. viridula* transcriptome. An overview of the BUSCO-based analysis for the *N. viridula* transcriptome are displayed. Fractions of single-copy (dark blue), multi-copy (light blue), fragmented (yellow), and missing (red) BUSCOs are shown. A) For the *N. viridula* transcriptome, the BUSCO analysis was run at the whole transcriptome, and also at the unigene set. The high BUSCO scores for the unigene set, together with the drastic reduction of duplicated BUSCOs compared to the whole transcriptome, show that it is suitable for for the downstream analyses, such as orthology and phylogeny analyses. B) The *N. viridula* unigene set was also compared against other available stink bug and Hemiptera gene sets. Importantly, all of the genomes and transcriptomes used in this work are of very good quality, as showed by the high BUSCO scores (> 80% complete BUSCOs).

**Additional file 3: Figure S3.** Breakdown of the lineage-specific *N. viridula unigenes.* The *N. viridula* unigenes without orthologs are divided into sub-categories depending on their blast hits and transcriptional activity. Overall, the majority of these lineage-specific unigenes were not transcribed, which is more evident in the the genes that do not have a blast hit.

**Additional file 4: Figure S4.** A photograph of the *N. viridula* midgut. The *N. viridula* dissected midgut is shown with labels for each of the 4 sections. The photo was taken under a light microscope after dissection in PBS buffer after the diet described in the current manuscript.

**Additional file 5: Table S1.** Transporter nomenclature comparisons. An equivalency table is shown indicating the nomenclature of transporter families and their proposed substrates. Transporters are generally named according to their SLC family, Transporter Classification Database code (e.g. 2.A.22) or their common name, which indicates their general function.

**Additional file 6: Table S2.** Proteomics Enrichment. A comparison of the number of proteins identified during different protein extraction methods is shown. As the pellet sample is thought to be enriched in transmembrane proteins, comparisons were made between proteins with predicted transmembrane domains and others. However, no difference in membrane proteins was found among the two techniques.

**Additional file 7: Table S3.** Transcript TPMs. A full table of all unigenes identified in this study with their predicted expression (average TPM values across 4 samples) are shown for each midgut compartment along with an annotation based on Uniref50. Corresponding sequences for transcripts can be found in the bioproject PRJNA557118.

**Additional file 8: Table S4.** Full proteome. A full table of all unigenes identified in this study with an indication of whether the unigene was detected in the proteome of a given tissue. Corresponding sequences for transcripts can be found in the bioproject PRJNA557118.

**Additional file 9: Table S5.** Other Stink Bug Transcriptomes. A table showing information regarding the other stink bug transcriptomes used in this study. A unigene set was obtained from the sequenced *H. halys* genome while the other three stink bugs were assembled de novo using the same pipeline that was used for *N. viridula*. Accession numbers can be found in column 3.

**Additional file 10: Table S6.** Gene Groups. A full list of all genes that were classified into one or more gene groups is shown. These groups were either derived from Fuzzy C-means clustering (Fig. 4) or from the top 500 most highly expressed genes in each tissue.

**Additional file 11: Table S7.** P450 Comparison. A table is shown which indicates the accuracy of the P450 identification pipeline used in the current study. A list of species with P450s already annotated (see references) were used as targets for the pipeline. The table also indicates the number of P450s predicted by our pipeline and the number previously shown in the literature.

**Additional file 12: Table S8.** P450 expression. A table is shown of all the P450s identified in our study. Corresponding information regarding sequence, P450 clan, name and expression values are also shown for each identified P450. NezVir_CYP6LV30 initially showed an extremely high TPM value (upwards of 2000), but manual inspection revealed this to be an artifact of the assembly, so it was excluded from the analysis.

**Additional file 13: Table S9.** Transporter expression. The full list of nutrient transporters and their corresponding expression values are shown. The table is broken down into transporters which act on amino acids/peptides and sugars. Corresponding information about the family to which the transporter belongs is also included.

**Additional file 14: Table S10.** BUSCO Scores. The Benchmarking Universal Single-Copy Orthologs (BUSCO) scores for each of the stink bug gene sets used in this study are shown. For each stink bug the differnet outputs of BUSCO are shown including, *Complete and single-copy*, *Complete and multi-copy*, *Fragmented*, and *Missing*.

**Additional file 15.** P450 sequences. All P450 sequences identified in this study are listed in fasta format.

**Additional file 16.** Transporter sequences. All transporter sequences identified in this study are listed in fasta format.

## Data Availability

The sequencing reads and de novo assembly are available from the Sequence Read Archive (SRA) under the bioproject accession PRJNA557118. Additional data and custom scripts (R, bash, and python) are available upon request.

## Abbreviations

AAAP: Amino acid and auxin permease
APC: Amino Acid-Polyamine-Organocation
GO: Gene ontology
NSS: Neurotransmitter:Sodium Symporter
POT: Proton-dependent Oligopeptide Transporter
SLC: Solute Carrier
SP: Sugar Porter
SSS: Solute:Sodium Symporter
TPM: Transcripts per kilobase million
