# Supplementary figures and images for: A transcriptomic and proteomic atlas of expression in the Nezara viridula (Heteroptera: Pentatomidae) midgut suggests the compartmentalization of xenobiotic metabolism and nutrient digestion
Source: BMC Genomics. 2020 Feb 6;21:129. doi: 10.1186/s12864-020-6459-6 (PMC7006211; doi:10.1186/s12864-020-6459-6)

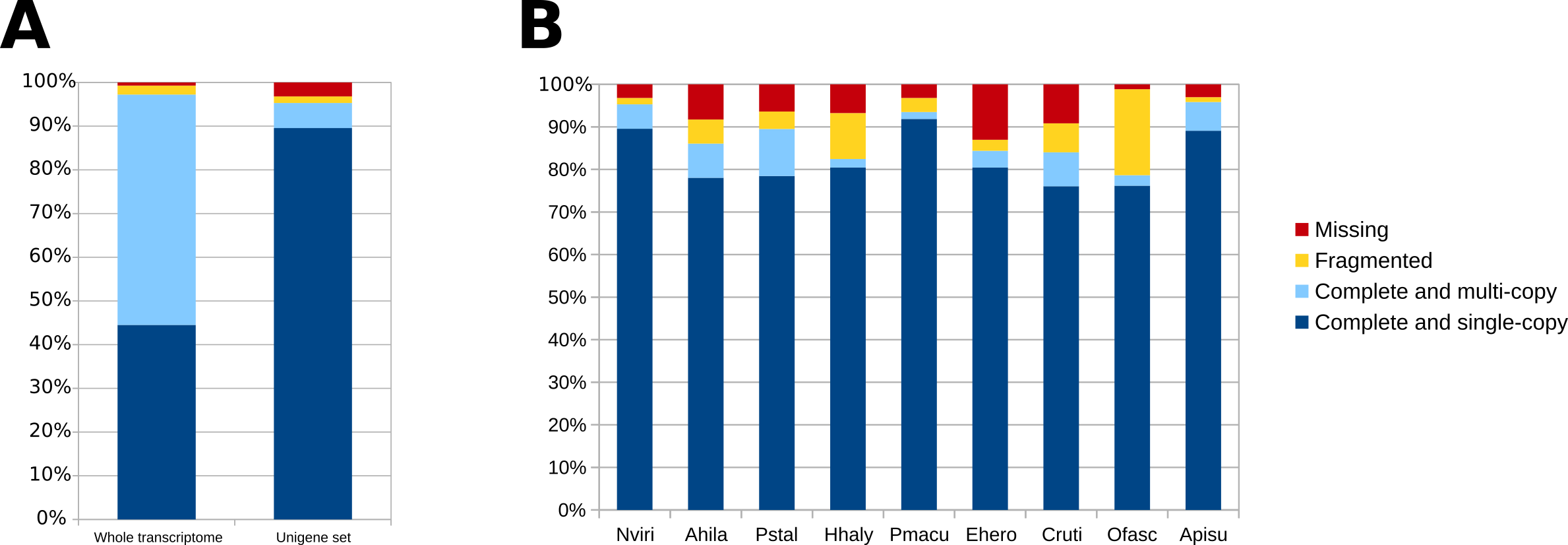

Supplement: Supplementary file 2 — Additional file 2: Figure S2. Quality of the N. viridula transcriptome. An overview of the BUSCO-based analysis for the N. viridula transcriptome are displayed. Fractions of single-copy (dark blue), multi-copy (light blue), fragmented (yellow), and missing (red) BUSCOs are shown. A) For the N. viridula transcriptome, the BUSCO analysis was run at the whole transcriptome, and also at the unigene set. The high BUSCO scores for the unigene set, together with the drastic reduction of duplicated BUSCOs compared to the whole transcriptome, show that it is suitable for for the downstream analyses, such as orthology and phylogeny analyses. B) The N. viridula unigene set was also compared against other available stink bug and Hemiptera gene sets. Importantly, all of the genomes and transcriptomes used in this work are of very good quality, as showed by the high BUSCO scores (> 80% complete BUSCOs). [file 12864_2020_6459_MOESM2_ESM.png]

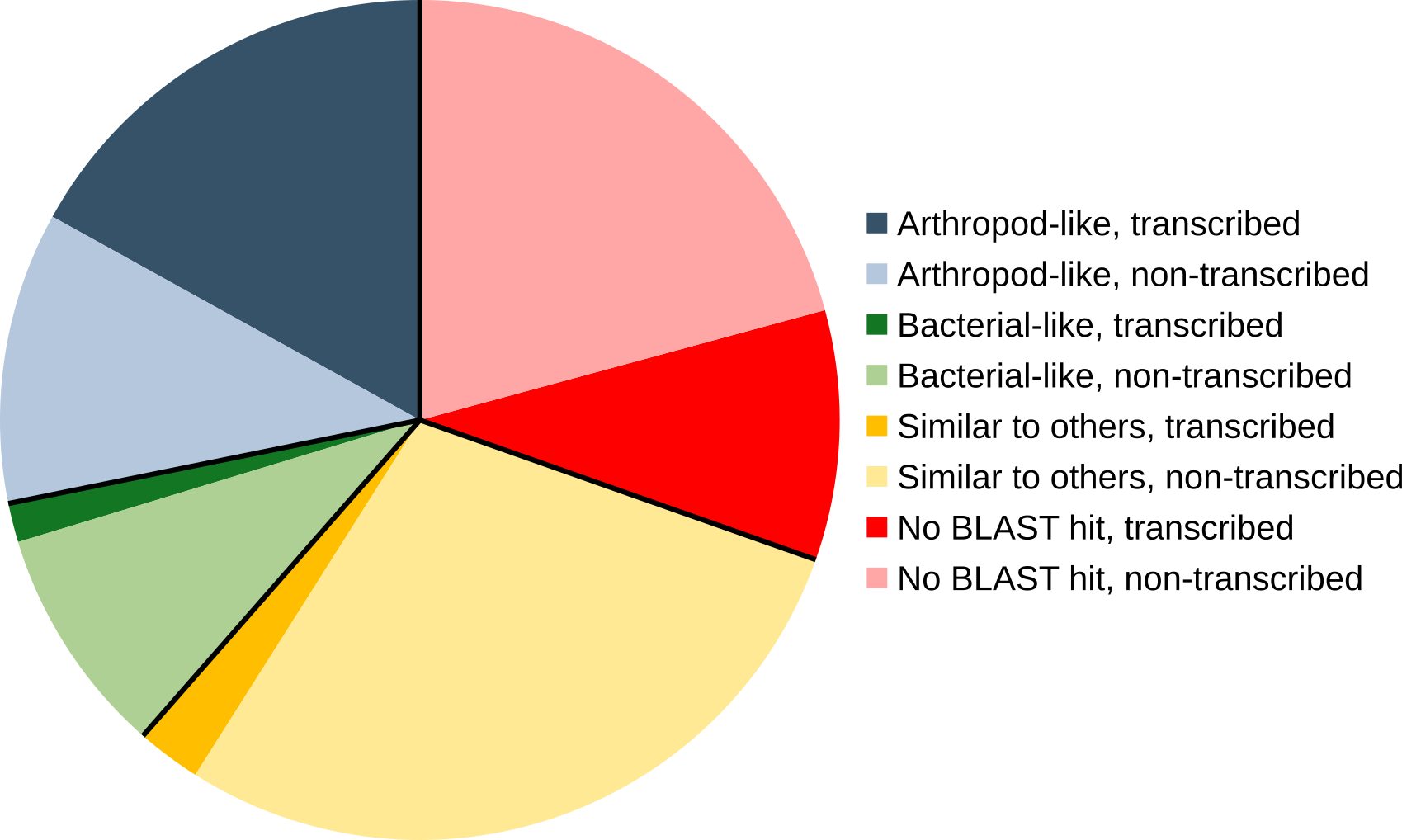

Supplement: Supplementary file 3 — Additional file 3: Figure S3. Breakdown of the lineage-specific N. viridula unigenes. The N. viridula unigenes without orthologs are divided into sub-categories depending on their blast hits and transcriptional activity. Overall, the majority of these lineage-specific unigenes were not transcribed, which is more evident in the the genes that do not have a blast hit. [file 12864_2020_6459_MOESM3_ESM.png]

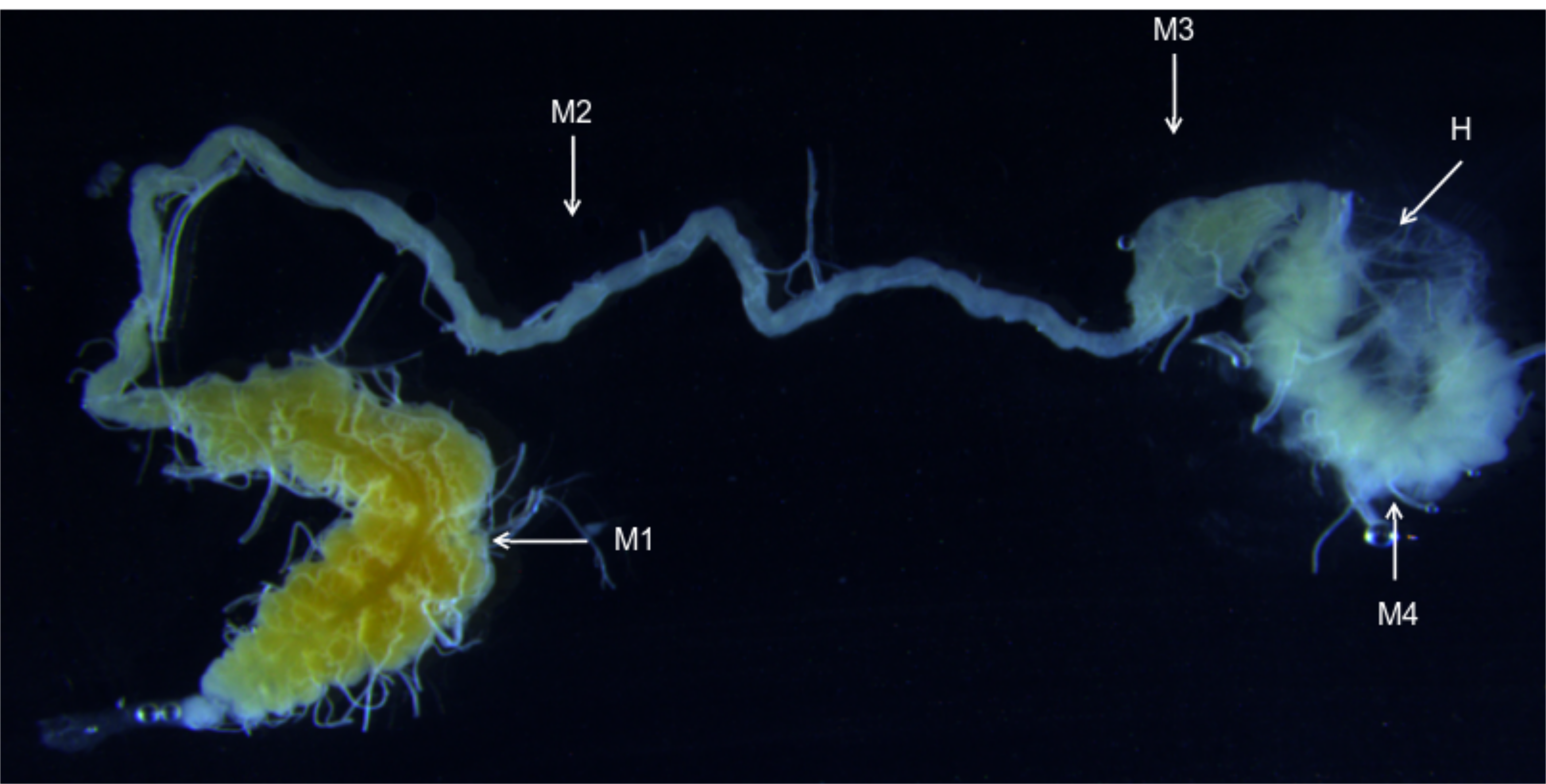

Supplement: Supplementary file 4 — Additional file 4: Figure S4. A photograph of the N. viridula midgut. The N. viridula dissected midgut is shown with labels for each of the 4 sections. The photo was taken under a light microscope after dissection in PBS buffer after the diet described in the current manuscript. [file 12864_2020_6459_MOESM4_ESM.png]
